# Urinary Protein Profiling for Potential Biomarkers of Chronic Kidney Disease: A Pilot Study

**DOI:** 10.3390/diagnostics12112583

**Published:** 2022-10-25

**Authors:** Abduzhappar Gaipov, Zhalaliddin Makhammajanov, Zhanna Dauyey, Zhannur Markhametova, Kamilla Mussina, Assem Nogaibayeva, Larissa Kozina, Dana Auganova, Pavel Tarlykov, Rostislav Bukasov, Zhandos Utegulov, Duman Turebekov, Maria Jose Soler, Alberto Ortiz, Mehmet Kanbay

**Affiliations:** 1 Department of Medicine, Nazarbayev University School of Medicine, Astana 010000, Kazakhstan; 2 Clinical Academic Department of Internal Medicine, CF “University Medical Center”, Astana 010000, Kazakhstan; 3 Department of Biomedical Sciences, Nazarbayev University School of Medicine, Astana 010000, Kazakhstan; 4 Department of Education, LLP BBNura, Astana 010000, Kazakhstan; 5 Department of Laboratory Diagnostics, National Scientific Medical Center, Astana 010000, Kazakhstan; 6 Department of Proteomics and Mass Spectrometry, National Center for Biotechnology, Astana 010000, Kazakhstan; 7 Department of Chemistry, SSH, Nazarbayev University, Astana 010000, Kazakhstan; 8 Department of Physics, SSH, Nazarbayev University, Astana 010000, Kazakhstan; 9 Department of Internal Medicine, Astana Medical University, Astana 010000, Kazakhstan; 10 Department of Nephrology, Vall d’Hebron University Hospital, Universitat Autònoma de Barcelona, 08193 Bellaterra, Spain; 11 Nephrology and Kidney Transplant Research Group, Vall d’Hebron Research Institute (VHIR), 08035 Barcelona, Spain; 12 Department of Medicine, Universidad Autonoma de Madrid and IIS-Fundacion Jimenez Diaz, 28040 Madrid, Spain; 13 Division of Nephrology, Department of Medicine, Koc University, 34450 Istanbul, Turkey

**Keywords:** urinary proteomics, proteinuria, chronic kidney disease, biomarkers

## Abstract

Proteinuria is a risk factor for chronic kidney disease (CKD) progression and associated complications. However, there is insufficient information on individual protein components in urine and the severity of CKD. We aimed to investigate urinary proteomics and its association with proteinuria and kidney function in early-stage CKD and in healthy individuals. A 24 h urine sample of 42 individuals (21-CKD and 21-healthy individuals) was used for mass spectrometry-based proteomics analysis. An exponentially modified protein abundance index (emPAI) was calculated for each protein. Data were analyzed by Mascot software using the SwissProt database and bioinformatics tools. Overall, 298 unique proteins were identified in the cohort; of them, 250 proteins belong to the control group with median (IQR) emPAI 39.1 (19–53) and 142 proteins belong to the CKD group with median (IQR) emPAI 67.8 (49–117). The level of 24 h proteinuria positively correlated with emPAI (r = 0.390, *p* = 0.011). The emPAI of some urinary proteomics had close positive (ALBU, ZA2G, IGKC) and negative (OSTP, CD59, UROM, KNG1, RNAS1, CD44, AMBP) correlations (r < 0.419, *p* < 0.001) with 24 h proteinuria levels. Additionally, a few proteins (VTDB, AACT, A1AG2, VTNC, and CD44) significantly correlated with kidney function. In this proteomics study, several urinary proteins correlated with proteinuria and kidney function. Pathway analysis identified subpathways potentially related to early proteinuric CKD, allowing the design of prospective studies that explore their response to therapy and their relationship to long-term outcomes.

## 1. Introduction

Chronic kidney disease (CKD) is a global public health concern that affects approximately 850 million people of the world population [1]. In 2017, CKD ranked 12th among the leading causes of death with more than 1.2 million deaths, and it is estimated that the all-cause mortality of CKD together with CKD-attributable cardiovascular diseases (CVD) reached over 2.6 million deaths in the same year [2]. These numbers are likely to progress, and by 2040, CKD may reach the 5th rank on the list of leading causes of global death [3]. Unfortunately, currently available treatments of CKD are not successful at improving disease outcomes due to the diagnosis of CKD mostly in the late stages. Therefore, early detection of CKD and understanding the mechanisms of its progression are probably the only effective strategy for preventing CKD-associated social economic burdens and mortalities.

Proteinuria is recognized as the most important marker and is a risk factor for CKD progression and associated complications among both diabetic and non-diabetic patients [4]. The presence of excess proteins in urine as proteinuria may reflect at least one of these following mechanisms, but is not limited to: increased blood levels of low-molecular-mass (LMM) proteins, such as immunoglobulin light chains (pre-renal proteinuria); a loss of selective filtration of high-molecular-mass (HMM) proteins (glomerular proteinuria); inadequate reabsorption of filtered LMM proteins (tubular proteinuria); loss of tubular cell-derived proteins (tubular proteinuria); loss of lower urinary tract-derived proteins (post-renal proteinuria) [4,5]. Among these, glomerular proteinuria and each tubular proteinuria independently and/or together are pathognomonic of renal damage [6].

Reabsorption of excess amounts of filtered proteins by proximal tubular epithelial cells (PTECs) has been reported to render toxic effects on PTECs leading to tubulointerstitial inflammation, tubular atrophy, interstitial fibrosis, and eventually, end-stage renal disease (ESRD) [5,7,8]. Thus, acceleration of CKD progression is highly expected in patients with higher proteinuria levels due to the tubular toxicity of filtered proteins. However, despite high levels of proteinuria, paradoxically, some patients may have a slow decline of renal function compared to those patients with lower levels of urinary protein excretion. Thus, there is a knowledge gap; why the severity of CKD progression could differ between patients with similar levels of proteinuria or some patients with higher proteinuria do not progress as rapid as expected? Differences in the physical-chemical properties of filtered proteins, the molecular weight (low, middle, and high molecular weight proteins), the biological activity or cell origin, or the stimuli for protein secretion of urinary proteins may underlie these differences [7,9].

Therefore, early detection of “high-risk candidate” proteins, which are associated with proteinuria and renal function, may serve as reliable diagnostic markers of CKD progression. Considering this knowledge gap, in this preliminary study, we aimed to compare urinary protein profiles and their relationship with levels of proteinuria and kidney function in CKD patients and healthy individuals. We also aimed to compare the biologic and pathophysiologic processes based on protein profiles of CKD patients and healthy individuals.

## 2. Materials and Methods

### 2.1. Patients and Study Settings

This cross-sectional pilot study included early-stage CKD patients with different levels of proteinuria, and 21 age and gender matched healthy subjects. The study was approved by Nazarbayev University Institutional Review Ethics Committee on 25 February 2020 (NU-IREC 208/06122019), and all participants were subject to sign an informed consent. This study is part of the trial that has been registered in ClinicalTrials.gov (Trial registration ID NCT04311684, the date of registration was 17 March 2020) [10]. Patient examinations were conducted according to good medical and laboratory practice and in keeping with the recommendations set forth by the Declaration of Helsinki Guidelines for Biomedical Research Involving Human Subjects.

Participants were recruited in this study at “National Scientific Medical Center” (NSMC, Astana, Kazakhstan) between March 2020 and December 2021 and were COVID-19-free at the time of enrollment. All participants were informed about the study protocol and gave informed consent prior to enrolling in the study. Inclusion criteria for the patient group were as follows: CKD stage 1–3 by KDIGO classification [11] caused by glomerular diseases, with proteinuria > 300 mg/24 h. Exclusion criteria were: age < 18 and >65 years, pregnant females, patients with diabetes mellitus, cancer, infectious diseases, and other life-treating comorbidities or conditions. The diagnosis of suspected glomerular disease was based on the presence of proteinuria in the absence of systemic diseases but was not confirmed by kidney biopsy.

### 2.2. Laboratory Tests and Data Collection

The laboratory investigators were blinded to data showing patients’ clinical outcomes until the end of the study. Blood and urine samples were collected independently, without considering the outcome of the patients. Blood metabolic profiles including serum creatinine, serum urea, glucose, uric acid, total protein, AST, ALT, lipid profiles, and protein fractions were obtained for all participants. A 24 h urinalysis including urine protein, sodium, potassium, uric acid, creatinine as well as complete blood count parameters was also defined during the enrollment. All routine laboratory tests were performed by colorimetric method on a Cobas Integra 400 plus analyzer (Oststeinbek, Germany). An estimated glomerular filtration rate (eGFR) was calculated via the CKD-EPI equation. Demographic data, medical history, and comorbid diseases were recorded after interviews with the participants and collected from medical carts.

Additional 24 h urine samples were immediately stored at −80 °C for further proteomic evaluation [12]. Acetone precipitation was used as a method for protein isolation as described by Sun et al. [13]. Final protein samples were resuspended in 25 mM NH4HCO3 and stored at −80 °C until use. Protein concentrations were measured with a NanoDrop 1000 (Thermo, Waltham, MA, USA) following the manufacturer’s instructions. A concentration of fifty μg of proteins was transferred to an Eppendorf tube for in-solution protein digestion.

### 2.3. Mass Spectrometry Analysis

The proteins were reduced, alkylated, and digested by trypsin (20 ng/uL) at 37 °C overnight, and the obtained peptide mixtures were desalted, and concentrated using ZipTip with C18 resin (Millipore, Cork, Ireland). The eluted peptides were then processed with a centrifugal evaporator (Eppendorf, Hamburg, Germany) and re-suspended in 10 μL of 0.1% trifluoroacetic acid. Samples were analyzed using nanoflow reversed-phase C18 liquid chromatography-tandem mass spectrometry (LC-MS/MS). For chromatography, a Dionex HPLC pump with a trapping column (Acclaim PepMap100 C18 pre-column) was used. An Acclaim PepMap100 C18 RSLC column (Thermo, MA, USA) was used to separate the peptides in a 75 min multistep acetonitrile gradient at a flow rate of 0.3 mL/min. An unmodified captive spray ion source (capillary 1500 V, dry gas 3.0 L/min, dry temperature 150 °C) was used with the Impact II ESI-QUAD-TOF mass spectrometer (Bruker Daltonics, Bremen, Germany). Full-scan MS spectra were acquired at a spectral rate of 2.0 Hz, followed by the acquisition of one MS/MS spectrum. The MS/MS data were analyzed in Data Analysis 3.4 software (Bruker Daltonics, Bremen, Germany) and saved in Mascot generic format (*.mgf) then searched with the Mascot 2.6.1 software (Matrix Science, London, UK) against the Swiss-Prot protein database (release February 2021) taxonomically restricted to *Homo sapiens* (human) containing 20,396 sequences. The search parameters used were oxidation of methionine as variable modification and carbamidomethylation of cysteine residues as fixed modification. A maximum of two missed cleavages of tryptic peptides was allowed. The peptide and protein data were extracted using a significance level of *p* < 0.05. The false discovery rate (FDR) was set to 1% and the decoy database search was used for estimating the FDR. Mass error windows of 100 ppm and 0.05 Da were allowed for MS and MS/MS, respectively.

Exponentially modified protein abundance index (emPAI) was selected as a label-free quantification technique based on spectral counting of tryptic peptides as described elsewhere [14]. The emPAI values were calculated for the identified proteins by the Mascot 2.6.1 software (Matrix Science, London, UK).

### 2.4. Reactome and GO Analysis

**Figure 1:**
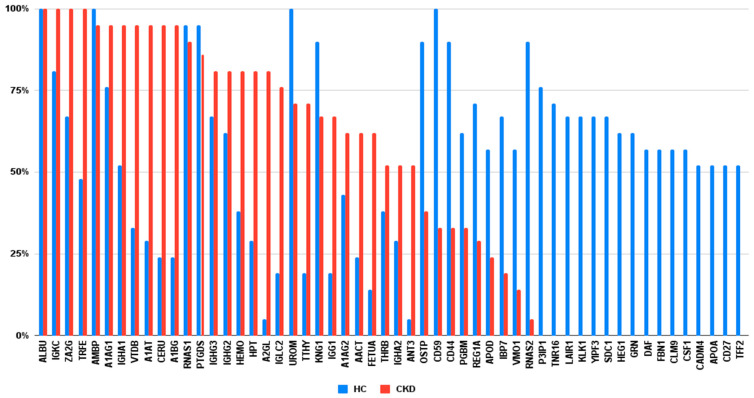
A comparison of protein distribution was identified in more than 50% of any group participants. CKD, chronic kidney disease; HC, healthy controls.

Highly abundant proteins were selected that were expressed in more than 50% of study participants to further analyze for functional enrichment and pathway analyses, which resulted in 29 proteins in the CKD group and 37 proteins in the control group. The list and percentage of the selected proteins identified in more than 50% of participants are presented in Figure 1. Gene Ontology (GO) enrichment analysis was conducted to gain functional insights for selected proteins, including biological process, protein class, and cellular components via Protein Analysis Through Evolutionary Relationships (PANTHER, http://pantherdb.org accessed on 18 March 2022) [15,16]. Pathway enrichment analysis was conducted using The Reactome Knowledgebase (https://reactome.org accessed on 18 March 2022) that produces a probability value (*p*-value) by correcting it for false discovery rates using the Benjamini–Hochberg method [17].

### 2.5. Statistical Analyses

Statistical analyses were conducted using Stata MP2 16.1 version. Numerical variables were expressed as mean ± standard deviation (SD) for normal distribution and median and interquartile range (IQR) for abnormal distribution. Categorical variables were presented as numbers (percentage), and for categorical variables, a chi-square test was performed. Two-sided *t*-tests for parametric and Wilcoxon rank-sum tests for nonparametric data were performed. Spearman’s correlation test was used for identifying correlations between the protein profiles and eGFR, a correlation between serum creatinine level with protein profiles, and correlations between the level of 24 h protein excretion and the emPAI of urinary proteins. All data were compared between healthy controls (HC) and CKD patients. A *p*-value less than 0.05 (typically ≤ 0.05) is considered statistically significant. The estimated power for the sample size of 42 participants was 0.9935.

## 3. Results

### 3.1. General Characteristics of the Study Population

The baseline characteristics of the study population are presented in Table 1. There are 21 healthy controls and 21 early-stage CKD patients comparable in terms of age, gender, and eGFR. Median eGFR was 109.1 (IQR 103.3–120.1) and 111 (72.6–138.1) mL/min in healthy control and CKD groups, respectively. In 90% of cases, the primary cause of CKD was glomerular diseases. The laboratory findings in the CKD group present the abnormal ranges related to glomerular disease (hypoproteinemia, hyperlipidemia, proteinuria), compared to healthy controls.

### 3.2. Urinary Protein Profiles of Study Population

Initially, 298 individual proteins were identified in the overall cohort (Table 1); of them, 250 belong to the control group with median (IQR) emPAI 39.1 (19–53) and 142 proteins belong to the CKD group with median (IQR) emPAI 67.8 (49–117). Figure 1 depicts that the observed selected proteome composition of urine samples markedly differs between groups. Most proteins with higher emPAI that were observed in urine samples of CKD patients were mostly found in a small number of urine samples of healthy individuals. Contrarily, most proteins that were observed in urine samples of healthy individuals were either not found or found in a small number of urine samples of patients. However, the amount of emPAI was significantly higher in the CKD group, corresponding to the amount of 24 h proteinuria.

### 3.3. Associations between Renal Function and Protein Composition

The level of 24 h proteinuria positively correlated with both the emPAI of total and selected proteins (r = 0.390, *p* = 0.011 and r = 0.532, *p* = 0.003, respectively). Table 2 shows the correlations between the level of 24 h protein excretion and the emPAI of the urinary proteome. The level of 24 h protein excretion positively correlated with ALBU, ZA2G, IGKC, FETUA, VTDB, A1BG, TRFE, and IGHA2, while the emPAI of OSTP, CD59, UROM, KNG1, RNAS1, CD44, AMBP, A2GL, and PTGDS proteins negatively correlated with the level of 24 h proteinuria.

The correlations of serum creatinine levels and eGFR with emPAI of some proteins shown in Table 3. The serum creatinine level positively correlated with VTDB (r = 0.617, *p* = 0.0008) and negatively correlated with AACT (r = −0.627, *p* = 0.007), A1AG2 (r = −0.464, *p* = 0.03), VTNC (r = −0.60, *p* = 0.04), whereas eGFR is positively correlated with CD44 (r = 0.470, *p* = 0.02) and negatively correlated with VTDB (r = −0394, *p* = 0.05). Overall, the most consistent correlation with markers of kidney function was provided by VTDB, as it correlated with both serum creatinine (positively) and eGFR (negatively).

### 3.4. GO Enrichment Analysis of Urinary Proteome

**Figure 2:**
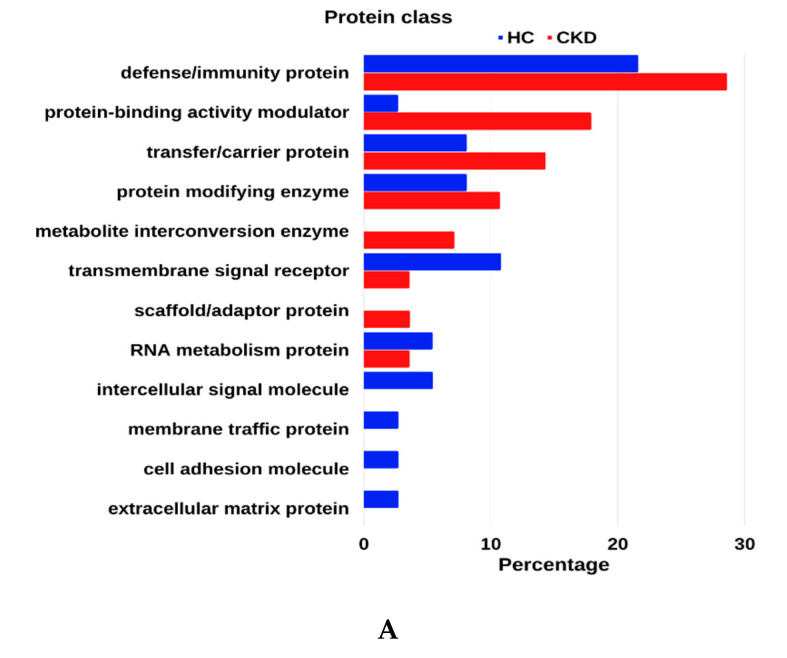

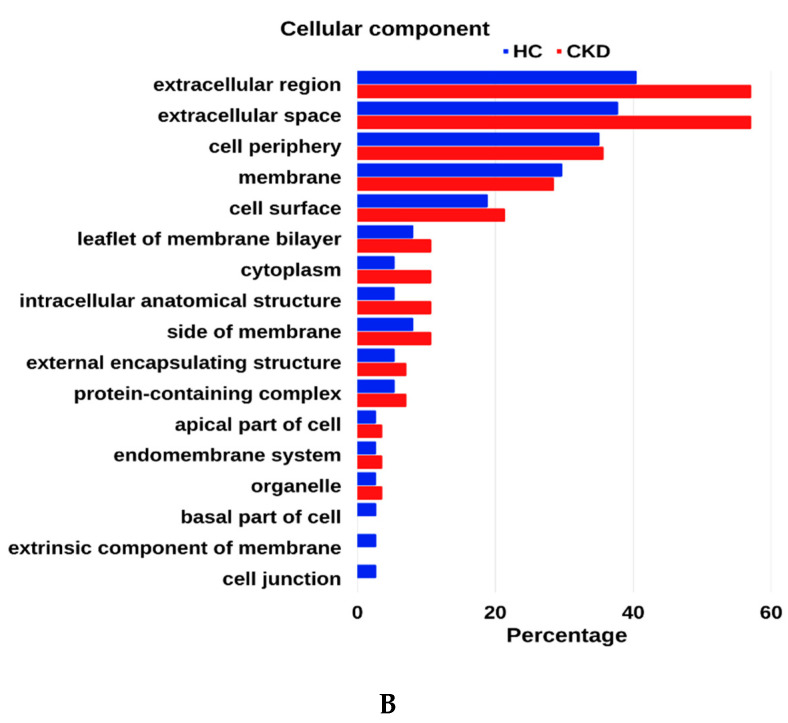

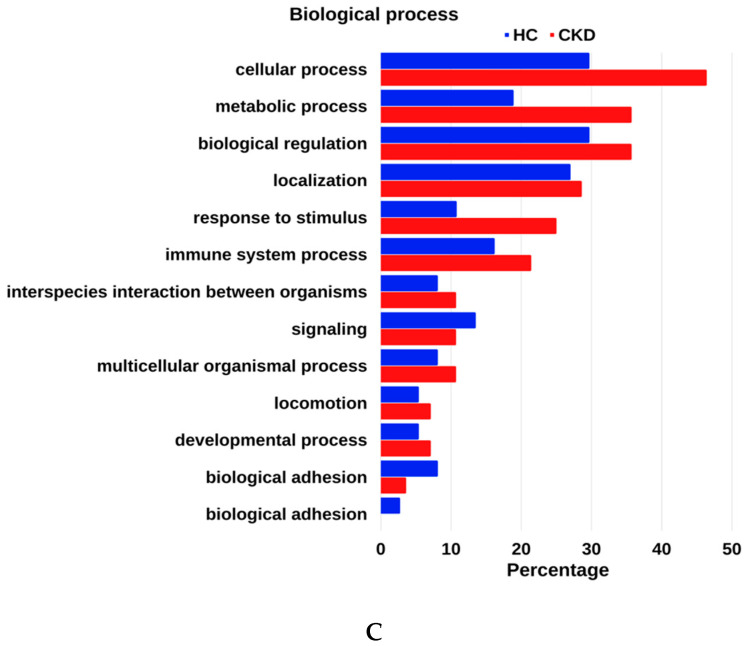
GO enrichment analysis of the 29 selected proteins in the CKD group (in red) and 37 proteins in the control group (in blue). (**A**) protein class. (**B**) cellular component. (**C**) biological process.

Figure 2 illustrates the GO enrichment analysis of selected proteins in the CKD and control groups. The protein class of GO analysis shows significantly higher numbers of proteins in the defense protein, protein-binding activity modulator, transfer protein, protein modifying enzyme, and metabolite interconversion enzyme classes in urine samples of the CKD group compared to urine samples of the healthy group. However, protein numbers in the transmembrane signal receptor, RNA metabolism protein, intercellular signal molecule, membrane traffic, cell adhesion, and extracellular matrix protein classes are higher in the urine samples of the controls compared to the urine samples of the CKD group (Figure 2A).

Further, the cellular component displays how most urine proteins are excreted from the extracellular region, cell periphery, membrane, and cell surface components of both groups. Notably, proteins that are excreted from the extracellular region are markedly higher in the urine samples of the CKD group compared to the urine samples of the healthy group (Figure 2B). 

Enrichment of proteins that are involved in cellular processes, metabolic processes, biological regulation, the response to stimulus, and immune system processes is significantly higher in the urine samples of the CKD group, while enrichment of proteins in signaling and cell adhesion processes are slightly higher in the urine samples of the healthy group (Figure 2C).

### 3.5. Pathway Enrichment Analysis of Proteins

The Reactome Knowledgebase generated signal cascades for 28 out of 29 submitted proteins of the patient group, where 123 different pathways were hit by at least one of the relevant proteins (Supplement Figure S1). For the control group, The Reactome Knowledgebase generated signal cascades for 30 out of 37 submitted proteins, where 163 different pathways were hit by at least one of the relevant proteins (Supplement Figure S2). Supplement Tables S1 and S2 provide results for pathway analysis of the 28 most relevant proteins of the CKD group and 31 relevant proteins of the control groups with an indication of significance level (*p* < 0.05) and false discovery rate (FDR < 5%).

**Figure 3:**
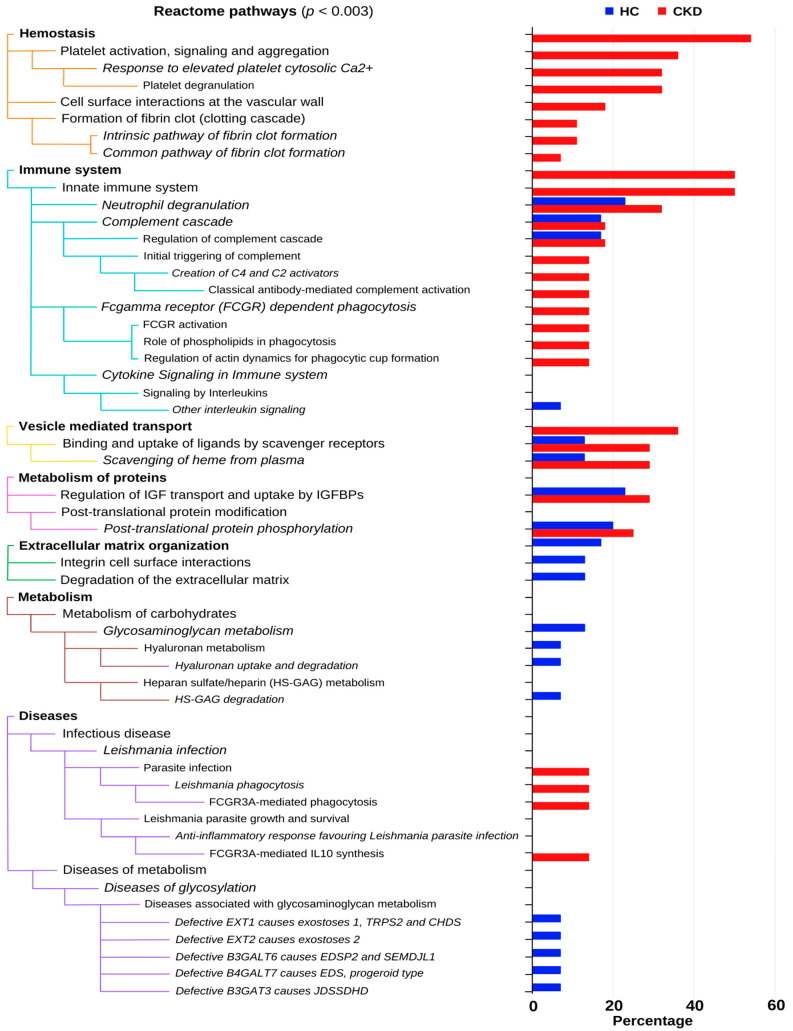
A comparison of highly significant pathways of the most relevant proteins in the CKD and healthy control groups (*p* < 0.003). The color codes denote the different pathway groups. CKD, chronic kidney disease; HC, healthy controls; IGF, insulin-like growth factor; IGFBPs, uptake by insulin-like growth factor binding proteins.

Further, to identify the most significant pathways in both groups, a filter was implicated using the significance level of *p* < 0.003. Figure 3 displays the comparison of the most significant pathways of 28 relevant proteins of the CKD group and 31 relevant proteins of the control groups. Pathway analysis shows that hemostasis, and the immune system and vesicle-mediated transport-related subpathways are significantly upregulated in participants with CKD compared to the healthy group. However, extracellular matrix organization-related pathways are downregulated in participants with CKD compared to the healthy group.

## 4. Discussion

This pilot study, assessing the urinary proteome in proteinuric patients with early GFR category CKD and healthy controls, found positive and negative relationships between emPAI levels of some urinary proteins with the amount of excreted 24 h proteinuria as well as with kidney function. Moreover, the differential presence of urinary proteins in CKD patients and healthy participants identified overrepresented biological processes in CKD patients, such as hemostasis, and immune system and vesicle mediated transport-related subpathways as well as, equally relevant, downregulated extracellular matrix organization-related pathways in participants with CKD compared to the healthy group. This information may be used to identify activated molecular pathways in CKD and assess the response to therapy.

Proteinuria usually results from a disrupted glomerular filter barrier, although it may also result from failure of the proximal tubular cells to reabsorb proteins filtered by healthy glomerular filter barriers or by excessive amounts of plasma proteins that are usually filtered by glomeruli (e.g., monoclonal light chains) [18]. Excess glomerular filtration of proteins causes tubular cell stress as tubular cells increase protein reabsorption, resulting in an inflammatory and profibrotic response and the loss of tubular cells. In routine clinical practice, only a few specific urinary proteins are measured for diagnostic purposes, including urinary albumin and kappa/lambda light chains. However, this approach potentially misses important information that may be provided by assessing a more ample protein panel. In this regard, the urinary peptidome may predict CKD progression and the plasma proteome at the time of active COVID-19 was recently found to associate with persistent symptoms out to 12 months [19,20,21].

In our study, we found statistically significant linear correlation between 24 h urinary protein level and the emPAI of albumin, zinc-alpha-2-glycoprotein, and immunoglobulin kappa constant. As expected, albumin (molecular weight 69 kDA) had the highest emPAI and highest relation to proteinuria. This finding is in correspondence with other literature data [22,23,24]. On the other hand, the emPAI of a few proteins with relatively higher molecular weight (>69 kDA), such as uromodulin, CD44 antigen, and kininogen-1 as well as with relatively lower molecular weight (<39 kDA), such as AMBP, osteopontin, CD55 glycoprotein and ribonuclease pancreatin were negatively correlated with the level of 24 h proteinuria. Uromodulin, which is exclusively produced by tubular cells [25], in contrast to albumin, may be down released from injured tubules in patients with higher proteinuria [26]. At this stage, it is difficult to explain the correlations between 24 h proteinuria and other proteins due to some limitations of this study, which needs to evaluate these relationships in urine samples, exclusively in a large cohort of CKD patients with proteinuria.

Our data showed a close relation between a few urine proteins and kidney function. Urinary Vitamin D-binding protein (VTDB) was consistently increased when kidney function declined. VTDB is the primary carrier in the circulation of 25-hydroxyvitamin D (25(OH) D), the precursor of the active form of vitamin D [27]. Urinary VTDB had previously been reported to predict kidney injury in patients with acute decompensated heart failure [28]. Other proteins were associated with at least some measures of kidney function. A1AG2 (orosomucoid) was upregulated in idiopathic nephrotic syndrome and diabetic nephropathy and associated with disease progression [29,30,31]. AACT, a serine protease inhibitor, also termed SerpinA3, is mainly synthesized in the liver and protects against different inflammatory diseases [32]. Urinary AACT levels were positively correlated with preclinical renal fibrosis following AKI [33]. Similarly, VTNC, an extracellular matrix protein secreted in response to tissue injury, was also expressed in preclinical renal fibrosis [34,35,36]. CD44 protein is a transmembrane protein, expressed by injured proximal tubular and glomerular cells [37,38,39]. The correlation between the abundance of these proteins with kidney function argues for their role in kidney disease.

We found an interesting difference in the biological process between CKD patients and healthy controls in gene ontology and Reactome enrichment analysis. Urinary proteins that function in the extracellular region were significantly higher in the urine samples of patients compared to controls, indicating increased permeability of the glomerular barrier, which allows the leakage of excessive serum proteins into the urine [5]. This is a hallmark of glomerular proteinuria in CKD, particularly in glomerulonephritis [40]. Importantly, the urinary composition of urine samples of the two groups significantly differs. Thus, GO and Reactome analysis show different biological process and pathway enrichment levels. In pathway enrichment analysis, subpathways of hemostasis, such as platelet activation, signaling and aggregation, cell surface interactions at the vascular level, and formation of a fibrin clot may represent the activation of platelets at the site of glomerular damage and their contribution to renal inflammation via interaction with leukocytes [41,42]. Previously, platelet–leukocyte aggregates were reported as a potential biomarker for kidney diseases and outcomes [42]. Upregulation of an immune system related subpathway, specifically neutrophil degradation, is likely due to acute inflammation during early stages of glomerulonephritis [43]. Activation of complement cascades was also overrepresented. Complement activation is an important mediator of pathogenesis and the progression to ESRD of multiple kidney diseases, including many types of glomerulonephritis [44]. Heme scavenging is also upregulated in CKD patients. In the context of glomerular disease, heme derived from the presence of red blood cells in the tubular lumen is a well-known tubular cell stressor that may cause tubular cell injury and death [45,46,47,48]. One more important point to note is the absence of extracellular matrix organization-related pathways in CKD patients. In this regard, proteinuria promotes tubular cell injury and interstitial inflammation and fibrosis [49]. A similar phenomenon had been previously observed using a related technique, urine capillary electrophoresis–mass spectrometry (CE-MS) peptidomics [19,20]. CKD273 is a urinary peptidomics biomarker composed of 273 peptides that differentiates CKD from healthy subjects and predicts CKD progression. Interestingly, many of the urinary peptides in CKD273 are type 1 collagen peptides and they are decreased in the urine of CKD subjects, suggesting that decreased ECM degradation could contribute to the accumulation of ECM during kidney fibrosis [19,20].

There are several limitations in our study. First, only patients with early-stage CKD (median eGFR 111 mL/min/1.73 m^2^) were enrolled and the study was cross-sectional. An ongoing follow-up study will address whether the current findings are associated with long-term kidney outcomes. Second, the sample size is limited. A multicenter study with a larger sample size is needed for validation of the current findings. However, the design of such a large multicenter study should be necessarily based on a pilot study, such as the present one. Third, the absence of kidney biopsy precludes the study of urine-tissue correlations.

## 5. Conclusions

This urinary proteomics study identified clear differences in the number and type of urinary proteins between proteinuric CKD patients with preserved kidney function and healthy individuals. Several urinary proteins were strongly correlated with 24 h proteinuria and/or kidney function. Interestingly, pathway analysis suggested significant upregulation of hemostasis, immune system, and vesicle mediated transport-related subpathways and decreased ECM subpathways in early stages of CKD. This information may be used to identify potential molecular mechanisms of kidney injury and to assess the impact of therapy on these mechanisms. Further prospective research is recommended with a larger study population to address both the impact of therapy on the urinary proteome and whether this proteomic pattern is an early biomarker of the response to therapy or of CKD progression.

## 6. Trial Registration

The trial was approved by ClinicalTrials.gov (Trial registration ID NCT04311684). The date of registration was 17 March 2020.

## Figures and Tables

**Table 1:** General and clinical biochemical characteristics of patients.

Parameters	Healthy Controls, *n* = 21	CKD Group, *n* = 21	*p*-Value
Demographics and clinical data			
Gender, female, %	15 (71%)	12 (57%)	0.334
Age, year	35.6 ± 8.3	38.5 ± 13.7	0.419
eGFR, mL/min/1.73 m^2^	109.1 (103.3–120.1)	111 (72.6–138.1)	0.835
Primary diagnosis, *n* (%)			
Glomerular disease	N/A	19 (90%)	
Post-transplant CKD	N/A	1 (4,76%)	
CKD of unknown etiology	N/A	1 (4.76%)	
Biochemistry			
Serum creatinine, µmol/L	65.1 (59.6–71.4)	69.6 (57.4–127.9)	0.285
Serum urea, mmol/L	4.0 (3.5–4.6)	5.2 (4.0–8.6)	0.014
Serum glucose, mmol/L	5.1 (4.9–5.5)	5.0 (4.4–5.3)	0.973
Serum uric acid, mcmol/L	277.5 (234.7–321.0)	359.6 (283.0–436.4)	0.018
Serum sodium, mmol/L	139.5 (138–141)	140 (139–141)	0.863
Serum potassium, mmol/L	4.4 (4.1–4.8)	4.2 (4.0–4.6)	0.232
Ca ionized, mmol/L	1.3 (1.3–1.3)	1.3 (1.3–1.3)	0.071
Total bilirubin, mcmol/L	7.1 (5.0–11.5)	5.0 (3.1–7.7)	0.042
ALT, mckat/L	0.2 (0.1–0.3)	0.3 (0.2–0.5)	0.111
ACT, mckat/L	0.3 (0.2–0.3)	0.3 (0.2–0.4)	0.396
Total cholesterol, mmol/L	4.4 (4.0–4.7)	6.5 (5.5–7.9)	<0.0001
HDL, mmol/L	1.5 (1.2–1.9)	1.4 (1.1–1.7)	0.514
LDL, mmol/L	2.7 (2.2–3.2)	4.1 (3.3–5.6)	0.0001
Triglycerides, mmol/L	0.9 (0.6–1.3)	2.3 (1.4–3.0)	<0.0001
Serum total protein, g/L	68.9 (66.5–72.0)	50.8 (44.8–64.6)	<0.0001
Protein fractions, %			
Albumin	68.4 (66.1–69.7)	58.0 (43.9–63.1)	<0.0001
Globulin alpa-1	1.9 (1.8–2.1)	2.9 (2.3–3.4)	<0.0001
Globulin alpa-2	8.5 (8.0–8.9)	14.3 (10.9–19.9)	<0.0001
Globulin beta-1	6.0 (5.6–6.4)	8.5 (7.1–9.7)	<0.0001
Globulin beta-2	3.2 (3.1–3.7)	5.4 (3.9–7.3)	0.0001
Gamma globulin	12.3 (10.9–14.4)	10.3 (8.5–13.8)	0.039
CBC			
WBC 10 × 10^9^/L	5.4 (5.0–6.2)	7.3 (6.8–8.9)	0.0003
RBC 10 × 10^12^/L	4.7 (4.3–5.2)	4.4 (4.0–4.7)	0.043
HGB g/L	129 (121–155)	125 (115–138)	0.220
PLT 10 × 10^9^/L	252 (228–298)	284 (228–329)	0.410
ESR, mm/h	9 (5–15)	27 (12–37)	0.002
Urinalysis, 24 h			
Proteinuria, g/24 h	0.1 (0.1–0.2)	2.0 (1.4–6.9)	<0.0001
Sodium, mmol/24 h	119 (71.0–164.9)	206.4 (145.0–317.1)	0.009
Potassium, mmol/24 h	40.8 (33.8–49.5)	42.2 (33.5–51.1)	0.697
Uric acid, mmol/24 h	2.4 (2.0–3.3)	2.6 (1.6–3.4)	0.990
Creatinine, mmol/24 h	9.6 (8.5–10.5)	9.7 (8.0–13.5)	0.464
Proteomic data			
Total # of defined proteins, *n*	250	142	
emPAI for total proteins	39.1 (19–53)	67.8 (49–117)	0.003
# of selected proteins, *n*	45	32	
emPAI for selected proteins	43.4 (24–71)	45.1 (18–80)	0.0001

Abbreviations: CKD: Chronic kidney disease; eGFR: estimated glomerular filtration rate; emPAI: exponentially modified protein abundance index; ALT: Alanine Aminotransferase; ACT: Activated Clotting Time; HDL: high-density lipoprotein; LDL: low-density lipoprotein; CBC: complete blood count; WBC: white blood cells; RBC: red blood cells; HGB: hemoglobin; PLT: platelet; ESR: Erythrocyte Sedimentation Rate.

**Table 2:** Correlations between the level of 24 h Protein excretion and the emPAI of Urinary Proteome.

Urinary Proteome	Protein Name	Spearman’s Rho	*p*-Value	N of Obs	Sum of emPAI	emPAI, Median (IQR)	Molecular Weight (Da)
ALBU	Albumin	0.6287	<0.0001	42	1388.9	21.5 (5.0–44.1)	69,367
ZA2G	Zinc-alpha-2-glycoprotein	0.6254	0.0001	35	37.32	1 (0.4–1.85)	34,259
IGKC	Immunoglobulin kappa constant	0.4199	0.0087	38	245.0	3.3 (1.1–6.2)	11,765
FETUA	Alpha-2-HS-glycoprotein	0.5347	0.0328	16	4.06	0.2 (0.1–0.4)	39,341
VTDB	Vitamin D-binding protein	0.4742	0.0125	27	13.67	0.3 (0.2–0.8)	52,918
A1BG	Alpha-1B-glycoprotein	0.4296	0.0321	25	25.85	1.1 (0.7–1.3)	54,254
TRFE	Serotransferrin	0.3815	0.0342	31	83.71	2.5 (0.7–4.0)	77,064
IGHA2	Immunoglobulin heavy constant alpha 2	0.4833	0.0494	17	11.13	0.6 (0.5–0.8)	36,591
OSTP	Osteopontin	−0.8017	<0.0001	27	9.47	0.3 (0.2–0.4)	35,423
CD59	CD59 glycoprotein	−0.7633	<0.0001	28	85.42	2.4 (1.3–4.1)	14,177
UROM	Uromodulin	−0.7589	<0.0001	36	56.16	1.1 (0.2–2.7)	69,761
KNG1	Kininogen-1	−0.7269	<0.0001	33	21.01	0.6 (0.1–1.0)	71,957
RNAS1	Ribonuclease pancreatic	−0.6924	<0.0001	39	38.62	0.6 (0.4–1.7)	17,644
CD44	CD44 antigen	−0.6803	0.0001	26	2.56	0.1 (0.04–0.1)	81,538
AMBP	Protein AMBP	−0.5899	<0.0001	41	160.57	1.9 (0.8–4.4)	38,999
A2GL	Leucine-rich alpha-2-glycoprotein	−0.5478	0.0186	18	4.60	0.2 (0.2–0.3)	38,178
PTGDS	Prostaglandin-H2 D-isomerase	−0.3365	0.0389	38	44.05	1.2 (0.5–1.3)	21,029

**Table 3:** Correlations of Serum Creatinine level and eGFR with emPAI of some proteins.

Information about Proteins				Serum Creatinine		eGFR	
Urinary Proteome	Protein Name	Molecular Weight (Da)	N of Obs	Spearman’s Rho	*p*-Value	Spearman’s Rho	*p*-Value
VTDB	Vitamin D-binding protein	52,918	26	0.6172	0.0008	−0.3944	0.0461
AACT	Alpha-1-antichymotrypsin	47,651	17	−0.6271	0.0071	0.3611	0.1544
A1AG2	Alpha-1-acid glycoprotein 2	23,603	21	−0.4637	0.0342	0.2023	0.3791
VTNC	Vitronectin	54,306	12	−0.5999	0.0392	0.4888	0.1068
CD44	CD44 antigen	81,538	25	−0.1365	0.5154	0.4696	0.0179

## Data Availability

The datasets produced after the completion of the final trial may be requested from the principal investigator (A.G.).

## Supplementary Materials

The following supporting information can be downloaded at: https://www.mdpi.com/article/10.3390/diagnostics12112583/s1, Figure S1: Pathway overview of the 29 most relevant proteins of the CKD group; Figure S2: Pathway overview of the 30 most relevant proteins of the healthy group; Table S1: Pathway analysis of the 28 most relevant proteins of the CKD group with the indication of significance level and false discovery rate; Table S2: Pathway analysis of the 30 most relevant proteins of the healthy group with the indication of significance level and false discovery rate.
