# Supplementary material for: Urinary Protein Profiling for Potential Biomarkers of Chronic Kidney Disease: A Pilot Study
Source: Diagnostics (Basel). 2022 Oct 25;12(11):2583. doi: 10.3390/diagnostics12112583 (PMC9689510; doi:10.3390/diagnostics12112583)
Supplement: Supplementary file 1 [file diagnostics-12-02583-s001.zip › diagnostics-1948342-supplementary.pdf]

diagnostics MDPI

Supplementary materials

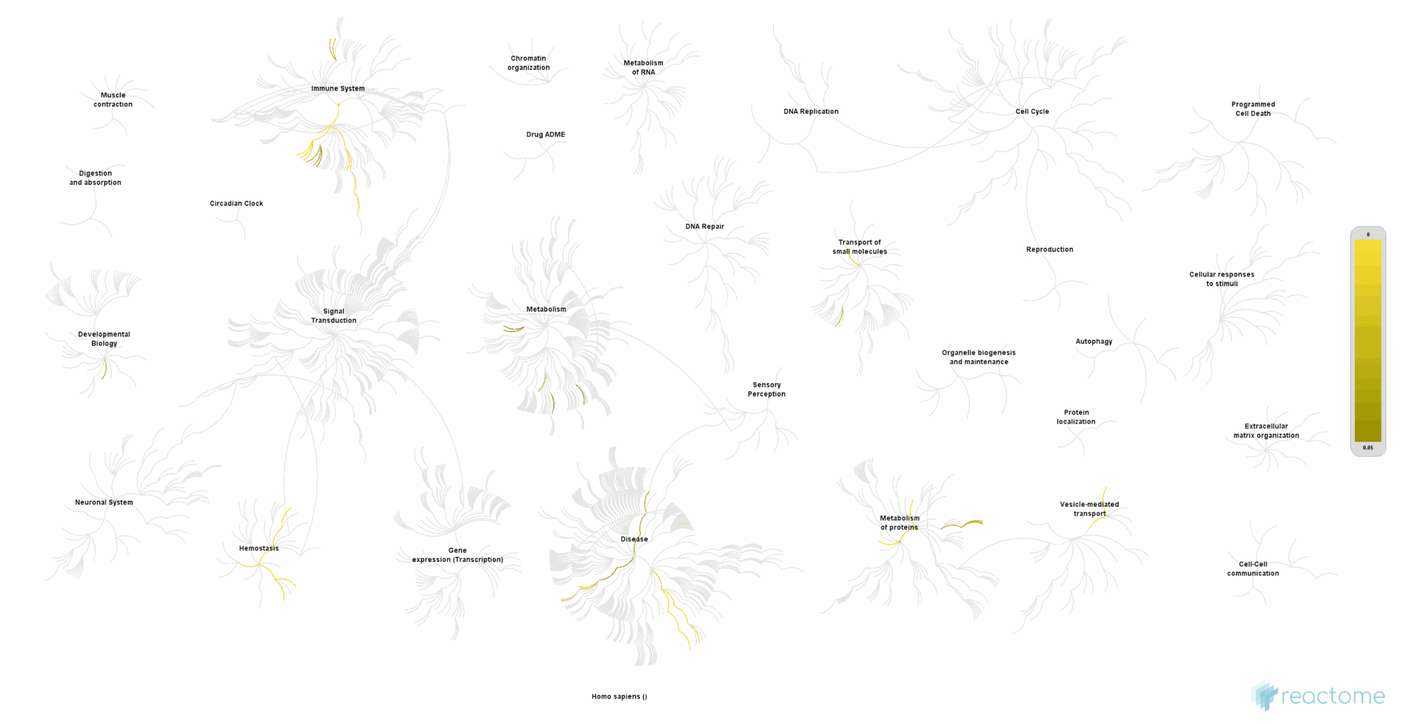

Scheme S1. Pathway overview of the 29 most relevant proteins of the CKD group. The color code yellow denotes the enrichment of the respective pathway with a generated significance level (p-value <0.05). The Reactome Knowledgebase, open-source, open access [17].

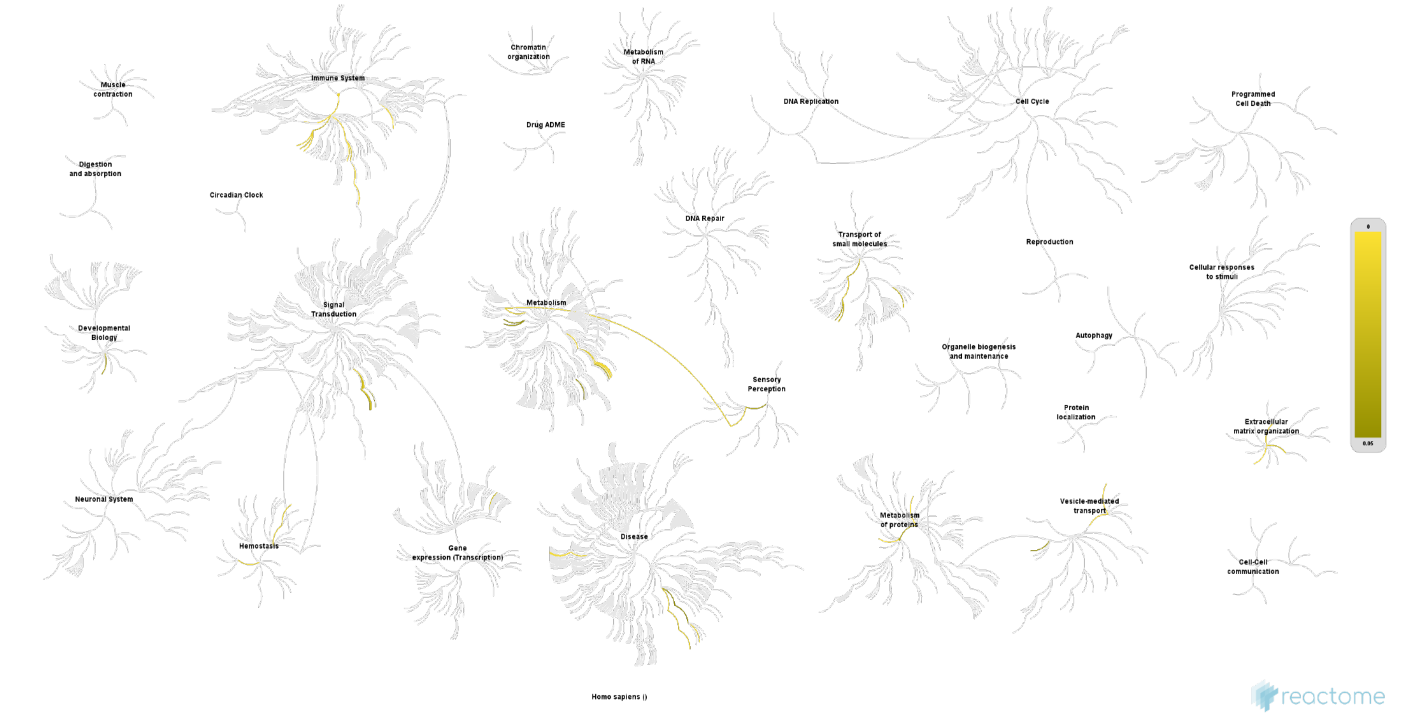

Scheme S2. Pathway overview of the 30 most relevant proteins of the healthy group. The color code yellow denotes the enrichment of the respective pathway with a generated significance level (p-value <0.05). The Reactome Knowledgebase, open-source, open access [17].

Table S1. Pathway analysis of the 28 most relevant proteins of the CKD group with indication of significance level and false discovery rate.

Pathway name	Identified proteins	Total proteins*	pValue	False discovery rate
Platelet degranulation	9	128	2.01E-11	1.26E-09
Response to elevated platelet cytosolic Ca2+	9	133	2.81E-11	1.26E-09
Hemostasis	15	726	2.99E-11	1.26E-09
Scavenging of heme from plasma	8	99	1.04E-10	3.22E-09
Platelet activation, signaling and aggregation	10	265	5.26E-10	1.27E-08
Regulation of Insulin-like Growth Factor (IGF) transport and uptake by Insulin-like Growth Factor Binding Proteins (IGFBPs)	8	124	6.03E-10	1.27E-08
Binding and Uptake of Ligands by Scavenger Receptors	8	129	8.21E-10	1.48E-08
Post-translational protein phosphorylation	7	107	7.47E-09	1.12E-07
Innate Immune System	14	1191	2.52E-07	3.53E-06
Neutrophil degranulation	9	480	1.69E-06	2.02E-05
Vesicle-mediated transport	10	761	9.33E-06	1.03E-04
Regulation of Complement cascade	5	135	2.02E-05	2.02E-04
Intrinsic Pathway of Fibrin Clot Formation	3	23	2.75E-05	2.47E-04
Complement cascade	5	146	2.93E-05	2.64E-04
Classical antibody-mediated complement activation	4	95	9.10E-05	7.28E-04
FCGR activation	4	101	1.15E-04	8.06E-04
Creation of C4 and C2 activators	4	103	1.24E-04	8.68E-04
Formation of Fibrin Clot (Clotting Cascade)	3	39	1.30E-04	9.13E-04
Initial triggering of complement	4	111	1.65E-04	9.90E-04
Role of phospholipids in phagocytosis	4	114	1.83E-04	0.001
FCGR3A-mediated IL10 synthesis	4	128	2.83E-04	0.0017
Cell surface interactions at the vascular wall	5	246	3.34E-04	0.0017
Immune System	14	2249	4.27E-04	0.002
Parasite infection	4	149	5.02E-04	0.002
Leishmania phagocytosis	4	149	5.02E-04	0.002
FCGR3A-mediated phagocytosis	4	149	5.02E-04	0.002
Regulation of actin dynamics for phagocytic cup formation	4	150	5.15E-04	0.0021
Fcgamma receptor (FCGR) dependent phagocytosis	4	175	9.13E-04	0.0036
Common Pathway of Fibrin Clot Formation	2	22	0.0014	0.0057
Anti-inflammatory response favouring Leishmania parasite infection	4	259	0.0038	0.015
Leishmania parasite growth and survival	4	259	0.0038	0.015

Defective SLC40A1 causes hemochromatosis 4 (HFE4) (macrophages)	1	2	0.005	0.015
Defective CP causes aceruloplasminemia (ACERULOP)	1	2	0.005	0.015
Post-translational protein modification	9	1423	0.0063	0.019
Defective F8 cleavage by thrombin	1	3	0.0075	0.022
Defective factor XII causes hereditary angioedema	1	3	0.0075	0.022
Iron uptake and transport	2	58	0.0093	0.028
Leishmania infection	4	345	0.01	0.031
CD22 mediated BCR regulation	2	70	0.013	0.04
Metabolism of proteins	10	1946	0.016	0.047
Defective factor VIII causes hemophilia A	1	7	0.017	0.047
Transport of gamma-carboxylated protein precursors from the endoplasmic reticulum to the Golgi apparatus	1	9	0.022	0.047
Antigen activates B Cell Receptor (BCR) leading to generation of second messengers	2	95	0.024	0.047
Removal of aminoterminal propeptides from gamma-carboxylated proteins	1	10	0.025	0.049
Gamma-carboxylation of protein precursors	1	10	0.025	0.049

* Number of total proteins in the Reactome Knowledgebase.

Table S2. Pathway analysis of the 30 most relevant proteins of the healthy group with indication of significance level and false discovery rate.

Pathway name	Identified proteins	Total proteins*	pValue	False discovery rate
Regulation of Insulin-like Growth Factor (IGF) transport and uptake by Insulin-like Growth Factor Binding Proteins (IGFBPs)	7	124	1.25E-07	2.11E-05
Post-translational protein phosphorylation	6	107	1.13E-06	9.46E-05
Regulation of Complement cascade	5	135	6.85E-05	0.0038
Complement cascade	5	146	9.88E-05	0.0041
Integrin cell surface interactions	4	85	1.58E-04	0.0052
Scavenging of heme from plasma	4	99	2.81E-04	0.0079
Neutrophil degranulation	7	480	7.19E-04	0.015
Binding and Uptake of Ligands by Scavenger Receptors	4	129	7.56E-04	0.015
Defective EXT1 causes exostoses 1, TRPS2 and CHDS	2	14	9.45E-04	0.015
Defective EXT2 causes exostoses 2	2	14	9.45E-04	0.015
Degradation of the extracellular matrix	4	140	0.001	0.015
Defective B3GALT6 causes EDSP2 and SEMDJL1	2	20	0.002	0.023
Defective B4GALT7 causes EDS, progeroid type	2	20	0.002	0.023
Defective B3GAT3 causes JDSSDHD	2	20	0.002	0.023
HS-GAG degradation	2	22	0.0023	0.024

Extracellular matrix organization	5	301	0.0026	0.024
Other interleukin signaling	2	24	0.0027	0.024
A tetrasaccharide linker sequence is required for GAG synthesis	2	26	0.0032	0.025
Innate Immune System	10	1191	0.0033	0.027
Classical antibody-mediated complement activation	3	95	0.0035	0.028
FCGR activation	3	101	0.0041	0.029
Creation of C4 and C2 activators	3	103	0.0043	0.03
HS-GAG biosynthesis	2	31	0.0045	0.031
Initial triggering of complement	3	111	0.0053	0.032
Plasma lipoprotein remodeling	2	35	0.0057	0.032
Role of phospholipids in phagocytosis	3	114	0.0058	0.032
Immune System	14	2249	0.0071	0.032
Glycosaminoglycan metabolism	3	124	0.0072	0.032
Hemostasis	7	726	0.0074	0.032
Cell surface interactions at the vascular wall	4	246	0.0077	0.032
Diseases associated with glycosaminoglycan metabolism	2	41	0.0077	0.032
FCGR3A-mediated IL10 synthesis	3	128	0.0079	0.032
Platelet degranulation	3	128	0.0079	0.032
Response to elevated platelet cytosolic Ca2+	3	133	0.0088	0.035
Retinoid metabolism and transport	2	44	0.0088	0.035
NFG and proNGF binds to p75NTR	1	3	0.0095	0.036
Ceramide signalling	1	3	0.0095	0.036
Metabolism of fat-soluble vitamins	2	48	0.01	0.036
Chondroitin sulfate/dermatan sulfate metabolism	2	50	0.011	0.036
Parasite infection	3	149	0.012	0.036
Leishmania phagocytosis	3	149	0.012	0.036
FCGR3A-mediated phagocytosis	3	149	0.012	0.036
Regulation of actin dynamics for phagocytic cup formation	3	150	0.012	0.036
Axonal growth stimulation	1	4	0.013	0.038
Heparan sulfate/heparin (HS-GAG) metabolism	2	55	0.013	0.04
Non-integrin membrane-ECM interactions	2	59	0.015	0.046

* Number of total proteins in the Reactome Knowledgebase.
